# microRNAs Orchestrate Pathophysiology of Breast Cancer Brain Metastasis: Advances in Therapy

**DOI:** 10.1186/s12943-020-1140-x

**Published:** 2020-02-15

**Authors:** Ranjana K. Kanchan, Jawed A. Siddiqui, Sidharth Mahapatra, Surinder K. Batra, Mohd W. Nasser

**Affiliations:** 1 Department of Biochemistry and Molecular Biology, University of Nebraska Medical Center, Omaha, Nebraska USA; 2 Department of Pediatrics, University of Nebraska Medical Center, Omaha, NE USA; 3 Eppley Institute for Research in Cancer and Allied Diseases, University of Nebraska Medical Center, Omaha, NE USA; 4 Fred and Pamela Buffett Cancer Center, University of Nebraska Medical Center, Omaha, NE USA

**Keywords:** Breast cancer brain metastasis, miRNA, Brain tumor microenvironment, Blood-brain barrier, CNS metastasis

## Abstract

Brain metastasis (BM) predominantly occurs in triple-negative (TN) and epidermal growth factor 2 (HER2)-positive breast cancer (BC) patients, and currently, there is an unmet need for the treatment of these patients. BM is a complex process that is regulated by the formation of a metastatic niche. A better understanding of the brain metastatic processes and the crosstalk between cancer cells and brain microenvironment is essential for designing a novel therapeutic approach. In this context, the aberrant expression of miRNA has been shown to be associated with BM. These non-coding RNAs/miRNAs regulate metastasis through modulating the formation of a metastatic niche and metabolic reprogramming via regulation of their target genes. However, the role of miRNA in breast cancer brain metastasis (BCBM) is poorly explored. Thus, identification and understanding of miRNAs in the pathobiology of BCBM may identify a novel candidate miRNA for the early diagnosis and prevention of this devastating process. In this review, we focus on understanding the role of candidate miRNAs in the regulation of BC brain metastatic processes as well as designing novel miRNA-based therapeutic strategies for BCBM.

## Introduction

Distant organ metastasis in breast cancer (BC) patients accounts for 90% of deaths [1]. In the central nervous system (CNS), the incidence of brain metastasis (BM) is ten times higher than primary brain lesions [2]. BC is the second most common cancer associated with BM with an incidence of BM, approximately 15-30% of total breast cancer cases [3]. Among different BC subtypes, triple-negative (TN) and HER2-positive BCs are more prone to BM [4]. Around 25% of BC cases show HER2 amplification, and out of these, 30-55% of patients develop BM with a median survival of only 4-14 months [5, 6]. Furthermore, TNBC patients with ER^-^/PR^-^/ HER2^-^ status are at high risk of BM recurrence [7].

Despite improvements in BC therapy, the treatment of patients with BM is still disappointingly challenging. BMs are commonly associated with poor prognosis and affect both cognitive and sensory functions of patients and limit the quality of life (QOL) [8]. Several markers, such as age, histology, ER/PR/HER2 status, and the number of non-CNS metastatic sites, have been used to predict BM from non-BM BC patients [9]. Owing to a high level of variability, these predictive markers have limitations.

In order to make progress in this field, there is an urgent need to improve the understanding of the pathobiology of BM, perhaps via first modeling the intricate process of metastasis in the brain microenvironment. The development of a BM is a multistep process, and the metastatic cellular niche is highly dynamic and heterogeneous [10, 11]. Moreover, the brain metastatic cell population harbors a unique genetic and epigenetic profile that distinguishes those cells from similar metastases in other organs [12]. Previous reports suggested an early onset of BM (22 months) after primary diagnosis with TNBC patients as compared to HER2+(30 months) and ER+/HER2^-^(63.5 months) BC patients [13]. Interestingly, the BBB protects the normal brain, looses its permeability partially or hetrogenously, and transforms into a blood-tumor barrier (BTB) which enhances the accessibily of therapeutic drugs to some extent, but not completly [14]. However the therapeutic role of BTB permeability is not well defined. Smith et al., demonstrated that BTB limits the uptake of chemotherapeutic drugs for BC, such as doxorubicin and paclitaxel into the brain relative to other organs [14, 15], suggesting limitations of BTB for the complete response of these drugs.

Growing evidence has demonstrated the role of miRNA in different steps of the metastatic process, including in BCBM, such as the epithelial to mesenchymal transition (EMT) [16–19], local invasion [20–23], intravasation [24–30], survival in circulation [31–33], extravasation [34], the integrity of the BBB [35–40], niche formation [41, 42], and colonization in the brain parenchyma [43–45]. MiRNAs are 20-22 short nucleotide sequences that often negatively regulate gene expression through the imperfect binding of their seed sequences to the 3’UTR region of target genes [46]. They can cleave or degrade target mRNA when binding with complete complementarity, and thereby inhibit translation of the target [47]. Recent investigations have revealed unique miR expression profiles in different cancer types at different stages, with compelling evidence supportive of miR-based staging and typing [48, 49]. In addition, miRNAs can regulate multiple genes and hence multiple processes simultaneously [50]. Given their ability to modulate the expression of multiple genes at a time, miRs are viewed as attractive therapeutic targets for cancer metastasis, a process mediated by multiple deregulated genes. This review discusses the functional role of miRNAs at different steps of BCBM in hopes of identifying novel miRNA-based therapeutic candidates for the treatment of this devastating process.

### Molecular events leading to BM: the role of miRNA

BM is a complex, multi-step, selective process. BM initiates by the dissemination of tumor cells from the primary site to the circulation and known as circulating tumor cells (CTCs). Prior to which these cells undergo EMT transition to invade the extracellular matrix (ECM) at the primary site. Then, to survive anoikis and immunosurveillance, primary tumor cells and CTCs secrete RNA and miRNA encapsulated in exosomes, which further facilitate the survival of metastatic cancer cells at the metastatic site. These miRNAs also transform brain stroma and breach the BBB for BM. Given the coordinated multi-step process that culminates in BM, miRs are perfectly poised to play a cardinal role in BM establishment, given their inherent ability to regulate multiple genes at a given time (Table 1). We have discussed below the role of miRNA at different stages of BM, starting from the primary site of dissemination to brain colonization.

**Table 1:** miRNAs mediated regulation of BCBM

miRNA	Targets	Regulation	References
EMT			
miR-8084	ING2, p53-BAX	upregulated	[16]
miR-484	PAX-5	upregulated	[19]
miR-708-3p	ZEB1, CDH2 and vimentin	downregulated	[17]
miR-210	E-cadherin (ORF), PAX-5	upregulated	[19]
miR-142-3p	Bach-1, CXCR4, MMP9, and VEGFR	downregulated	[18]
miR-199a/214	Slug	downregulated	[51]
miR-3178	Notch1	downregulated	[52]
miR-212-5p	Prrx2	downregulated	[53]
miR-29,miR-30 miR-200 family	ADAM12-L	downregulated	[54]
Intravasation			
miR-126	VEGF/PI3K/AKT axis, MAPK	downregulated	[30]
miR-520/373	ANGPTL4, PTHrP, PAI-1	downregulated	[25]
miR-204	ANGPT1 and TGFβR2	downregulated	[24]
miR-200 family	IL-8 and CXCL1	downregulated	[27]
miR-105	ZO-1	downregulated	[29]
Intravascular Microenvironment			
miR-141	Protection in circulation	upregulated	[31]
miR-183	DAP12/NK cells	downregulated	[32]
Extravasation in Brain Microenvironment			
miR-7, let-7c, miR-21	FasL, SERPIN1	upregulated	[55]
miR-200c	FAP-1	downregulated	[56]
miR-206	Cx43	downregulated	[57]
miR-19a, miR-32,miR-124a, miR-130b, miR-148a, and miR-583	PCTH7	downregulated	[58]
miR-125a/b-5p	ET-1	downregulated	[59]
miR-1266, miR-185 and miR-30c	BCL2L1	downregulated	[60]
miR-151-3p	TWIST1	downregulated	[61]
miR-17	ICAM-1and E-Selectin	downregulated	[62]
miR-126 and miR-1185	VCAM1	downregulated	[63]
miR-483-5p	ALCAM	downregulated	[64]
miR-21-3p	L1CAM	upregulated	[34]
miR-212	HBEGF	downregulated	[65]
miR-655	COX2	downregulated	[66]
miR-200b, 200c	ST6GALNAC5	downregulated	[67, 68]
BBB Regulation			
miR-181c	PDPK1	upregulated	[69]
miR-143	PUMA	upregulated	[35]
miR-125a-5p	ICAM-1	downregulated	[38]
miR-1258	HPSE	downregulated	[40]
miR-210	Occludin, β-catenin	upregulated	[37]
Cross Talk and Niche Formation			
miR-26a	PTEN ATM	upregulated	[70, 71]
miR-19a	PTEN	upregulated	[42]
miR-345	KISS1	upregulated	[72]
miR-124, miR-155, miR-689	Associated with M1 phenotype of microglia	upregulated	[73]
miR-711 and miR-145	Associated with M2 phenotype of microglia	upregulated	[73]
miR-503	L1CAM trigger M1–M2 polarization of microglia	upregulated	[41]
Metabolic Reprogramming			
miR-122	PKM2 , GLUT-1	upregulated	[74]
miR-155	PIK3R1-PDK/AKT-FOXO3a-cMYC axis	downregulated	[75]
miR-7	RelA	upregulated	[76]
Colonization			
miR-200 family (miR-200a,200b, 200c, miR-141, and miR-429)	ZEB1 and ZEB2	upregulated	[43, 44]
miR-147	ZEB1	upregulated	
miR-126	IGFBP2, PITPNC1 and MERTK	downregulated	[77]

#### miRNA-mediated activation of EMT

Although the EMT is both highly conserved and vital for normal developmental processes [78], it serves an essential role in metastasizing cancer cells [79]. In cancer pathogenesis, EMT promotes the dissemination of the primary tumor [80]. EMT transcription factors (TFs), such as TWIST1, SNAIL1, and SLUG, are contributory to BC metastatic potential and associated with poor prognosis [81]. ADAM12, a long splice variant with a transmembrane domain and member of the disintegrin and metalloproteinase family [82], can be induced by Twist1, thereby promoting tumor invasion via regulation of invadopodia formation and focal adhesions [83]. MiR-34a suppresses BC metastasis by downregulating EMT-TFs (SLUG, TWIST1, and ZEB1/2) and NOTCH1 signaling [81]. Further, ADAM12 is a direct target of the miR-29 and miR-200 families, both involved in BC progression [54]. Aside from regulating EMT-TFs, miRNAs can also regulate cytoskeletal rearrangement in cancer cells by targeting the expression of key molecules and cell signaling pathways involved in cell adhesion [84]. MiR-8084, miR-708-3p, miR-96-182-183 cluster, miR-484, miR-210, and miR-142-3p modulate the invasive potential of BC cells by modulating EMT [16–19]. Recently, it has been shown that miR-124, miR-199a/214, miR-3178, miR-30a, miR-508-3p and miR-212-5p can modulate the level of EMT markers and TFs regulating the expression of E-cadherin in TNBC, a subtype that commonly metastasizes to the brain [51–53, 85].

#### miRNA-mediated intravasation

Once breast tumor cells change their phenotype through EMT-driven mechanisms, metastasizing tumor cells start the process of metastasis by intravasation into nearby capillaries to facilitate neovascularization for survival [86]. To metastasize at distal sites, cancer cells begin contacting endothelial cells via adhesion molecules and protein receptors [86]. They then follow an amoeboid motility pattern and squeeze themselves between endothelial cells [87]. Some secretory miRNA can regulate the integrity of the endothelium, and thereby the process of intravasation. For instance, miR-105 that is secreted by BC cells disrupts the endothelium by targeting Zonula occludens protein-1(ZO-1), a tight junction protein1 (TJP-1) [29], thus promoting BM. Deryugina et al. discovered an alternative intravasation model suggestive of intravasation within the interior core of a primary tumor in parallel to stromal invasion [88].

Angiogenic factors and growth factors either released by tumor cells or stromal cells individually or during their mutual crosstalk contribute to intravasation [89]. These factors allow tumor cells to invade through the basement membrane, adhere to the endothelial membrane, and pass through endothelial gap junctions to disseminate into the circulation [86]. Although no miRNA has been reported to influence intravasation directly, they can regulate angiogenic signals by targeting angiogenic factors and protein kinases. A recent study discovered a novel role of TGF-β by tumor-associated fibroblasts (TAFs) in the organization of tumor blood capillaries. TAFs enhanced vessel coverage by pericytes, which are vascular cells that support capillaries [90]. In this regard, mRNA profiling of miR-520/373 overexpressing metastatic MDA-MB-231 cells elicited a strong downregulation of TGF-β signaling. It has also been reported that miR-520/373 are instrumental in reducing metastasis through downregulating TGF-β dependent potent angiogenic factors such as plasminogen activator inhibitor-1 (PAI-1), parathyroid hormone-related protein (PTHrP), and angiopoietin-like 4 (ANGPTL4) [25]. In a separate study, miR-204 was shown to suppress vascularization and angiogenesis *in vitro* and *in vivo* through targeting pro-angiogenic ANGPT1 and TGFβR2 in BC [24]. The miR-200 family could also play a role in regulating angiogenesis by directly targeting the pro-angiogenic cytokines IL-8 and CXCL1 in endothelial cells [27].

#### miRNA-mediated survival in the intravascular microenvironment

When a primary tumor grows, circulating tumor cells (CTCs) are shed and enter the circulation. Most CTCs are phagocytosed or undergo apoptosis, leaving behind only a few surviving CTCs to arrive at the targeted organ. Metastatic tumors, as well as CTCs from the primary tumor, may exhibit characteristics different from those of their cell of origin. In order to survive, CTCs must overcome anoikis and immune surveillance once they detach from the primary tumor. One of the tools exploited by CTCs after entering the circulation is platelet activation; by inducing platelet aggregation, tumor cells are protected from immune surveillance, undergo cell arrest within the vasculature, and experience enhanced survival [91, 92]. The CSCs phenotype of BC cells is associated with brain tropism in TNBC patients [93–95]. Debeb et al. have shown that overexpression of miR-141 in the MDA-MB-231 cell line enhances its brain tropism in a tail vein injection mouse model. Further, knockdown of miR-141 inhibited the metastatic ability of inflammatory BC to the brain, suggesting that miR-141 protects cells in the circulation and helps with colonization in the brain [31].

Platelets also contribute to immune evasion by CTCs from scavenging natural killer (NK) cells by enshrouding CTCs and releasing TGFβ and platelet-derived growth factor (PDGF) that directly inhibit the activity of NK cells [96]. Platelet-derived microparticles (PMPs) are major repositories for miRs, and platelets can transfer miRNA contents and modulate gene expression in CTCs [33]. PMP encapsulated miR-183 can suppress NK cell activation, possibly via the silencing of DAP12 a key accessory protein critical for surface NK receptor stabilization and downstream signal transduction [32]. Platelets also contribute to attenuate the early formulation of a metastatic niche [97]. Thus, platelet-derived miRNA also helps in the survival of CTCs after intravasation. The role of miRNA released by CTCs and the intravascular microenvironment in establishing a brain pre-metastatic niche formation warrants further investigation.

#### Extravasation

Once CTCs are able to survive in circulation, BC cells arrest in blood capillaries and start the process of extravasation, a process coordinated by many oncogenes [98]. Many pairs of ligand-receptor molecules contribute to the process of extravasation, including selectins, integrins, cadherins, CD44, and immunoglobulin superfamily receptors [99, 100].

Extravasation is a rate-limiting step for BCBM, as cancer cells must overcome the initial defenses imposed by astrocytes and other protective factors in the brain microenvironment [101]. Astrocytes that are mobilized to the metastatic brain lesion at a very early stage of colonization induce apoptosis through the FasL-mediated pathway [102]. In recent studies, several miRNAs have been described to target various members of the Fas-mediated apoptotic pathway. For example, miR-7, let-7c, and miR-21 regulate the expression of FasL [55], while miR-200c regulates the induction of apoptosis through CD95 by targeting FAP-1 [56]. Cancer cells release protease inhibitors known as serpins to combat the apoptotic effects exerted by astrocytes. MiR-21 has been shown to inhibit Serpin1, a gene with novel tumor-suppressive effects in gastric cancer [103]. However, its role in BM is unknown. Eventually, astrocytes support CTCs survival in brain parenchyma via establishing connexins (Cx) gap junctions and promote BM [104]. The expression of miR-206 is inversely correlated with Cx43 levels and is associated with decreased proliferation and migration [57]. PCDH7 in brain tropic BCs contributes to establishing Cx43 gap junctions with astrocytes and forms Ca^++^ channels [104]. A high PCDH7 level in the brain tropic CSC population has been reported and contributes to CSC extravasation, adaptation, and colonization in the new niche formation through the PCDH7-PLCb-Ca2þ-CaMKII/S100A4 pathway involving PCDH7-mediated tumor–astrocyte interaction [95]. In addition, miR-19a, miR-32, miR-124a, miR-130b, miR-148a, and miR-583 have been reported as potential regulators of PCDH7 [58]. However, the role of these miRNAs in PCDH7 regulated BM has yet to be studied [95].

The production of IL6 and IL-8 by cancer cells requires the establishment of gap junctions with astrocytes [105]. These cytokines influence both cell types by inducing the expression of endothelin ligand (ET-1) on astrocytes and endothelin receptors (ETAR and ETBR) on cancer cells [101, 105]. ET-1 is regulated through miR-125a/b-5p in endothelial cells [59]. In addition, the expression of a few genes was found to be dependent on such interaction [106]. Some of them were validated in BM, such as TWIST1, GSTA5, and BCL2L1 [106]. Interestingly, BCL2L1 is regulated by miR-1266, miR-185, and miR-30c [60] in prostate cancer. TWIST1 is regulated by miR-151-3p in BC [61]. These miRNAs are involved in negative regulation of the apoptotic pathway and upregulation of invasion or migration respectively, but the role in BM is not clear yet.

Emerging evidence shows that cell adhesion molecules (CAMs) play an essential role in extravasation through a cell-cell adhesion receptor. In an *in vivo* model of BM, a subset of adhesion molecules, including E-selectin, VCAM-1, ALCAM, ICAM-1, VLA-4, and a4 were found to be upregulated in the cerebral endothelium when injected intracardially. Conversely, the expression of their ligands (PSGL-1, VLA-4, ALCAM, LFA-1, and VCAM-1) was upregulated in brain tropic cancer cells [107], revealing a crucial role for these CAMs during the initial steps of extravasation. MiRNAs post-transcriptionally regulate CAMs. For instance, TGF-β induced ICAM-1, and E-selectin expression is regulated by miR-17 [62]. MiR-126 and miR-1185 regulate endothelial expression of VCAM1 [63, 108]. ALCAM is reported as a target gene of miR-483-5p [64].

Moreover, cancer cells can invade through the endothelium by projecting invadopodia [109]. Invadopodia are chemosensing protrusions that guide cancer cell extravasation to promote brain tropism in metastasis [110]. PAK1 (P21 (RAC1) Activated Kinase 1) is responsible for guiding cancer cell extravasation in BCBM [110]. PAK1 reduces the expression of miR-132 through the PAK1/ATF2/miR-132 axis. L1CAM, an adhesion molecule, mediates the spread of metastatic cells on the vasculature and additionally mediates interactions between cancer cells and endothelial cells in BM. The depletion of L1CAM in cancer cells fails to co-opt brain capillaries and hence is unsuccessful in promoting metastatic outgrowth. Interestingly, miR-21-3p was reported to be a positive regulator of L1CAM expression [34]. These studies strongly suggest that miRNAs can modulate the expression of various CAMs in cancer, as well as endothelial cells, and thereby play a decisive role in the establishment of metastasis at the distant metastatic site via extravasation.

Reactive astrocytes have been shown to contribute to the formation of a protumorigenic niche via a number of mechanisms involving secreted molecules. In the BCBM mouse model, Massague’s group has identified 17 genes that are specifically correlated with BC-metastasis associated genes. Among these 17 genes, four genes, COX2, EGFR ligand HBEGF, ANGPTL4, and the a2,6-sialyltransferase ST6GALNAC5 were identified as signature molecules of BC metastasis to the brain parenchyma [67]. COX2 is actively involved in BM by regulating the expression of MMP-1 in BC patients, and high expression is reported in BC patients [111]. Interestingly, COX2 expression is associated with BBB permeability . COX2 induces a stem-like cell phenotype by upregulating miR-655 and miR-526b in BC, thereby rendering cells more metastatic [66, 112]. MiR-212 directly targets HBEGF and suppresses cell growth, migration, and invasion [65]. ST6GALNAC5, a direct target of miR-200c, is a specific mediator of BCBM [67]. Conversely, the upregulation of ST6GALNAC5 in brain-tropic BC cells showed a decrease in adhesive properties of the endothelial component of a well-characterized human BBB *in vitro* model [113]. ST6GALNAC5 can also regulate the EMT process in BM and is a target of miR-200b [68]. Several target genes actively participate in extravasation within the brain parenchyma, although their regulation in context of miRNA is not studied in BCBM.

### Blood-brain barrier

The blood-brain barrier (BBB) is a semipermeable barrier comprised of endothelial cells, astrocytes, and pericytes, forming the neurovascular unit [114]. It remains important to study the role of miRNAs in enhancing the permeability of the BBB. Endothelial cells are interconnected with each other via tight junctions, a functionally important component of the BBB, controlling the free flow of substances into the brain parenchyma. Most of the solutes that are allowed to permeate the BBB, such as glucose, macronutrients, and electrolytes, enter via transporters present on the surface of endothelial cells.

Endothelial tight junctions facilitate the transmigration of tumor cells through the BBB [115]. CD44, VEGF, and CXCR4 contribute to the transendothelial migration process by disturbing endothelial integrity [116]. Astrocytes are indispensable for the development and maintenance of the BBB [106]. The intracellular junctions of brain endothelial cells form with tight junction proteins, such as occludin, claudins, and ZO-1 proteins [117]. Disruption of intercellular junctions causes the breakdown of the BBB and transform it into BTB [118, 119].

**Fig. 1.**
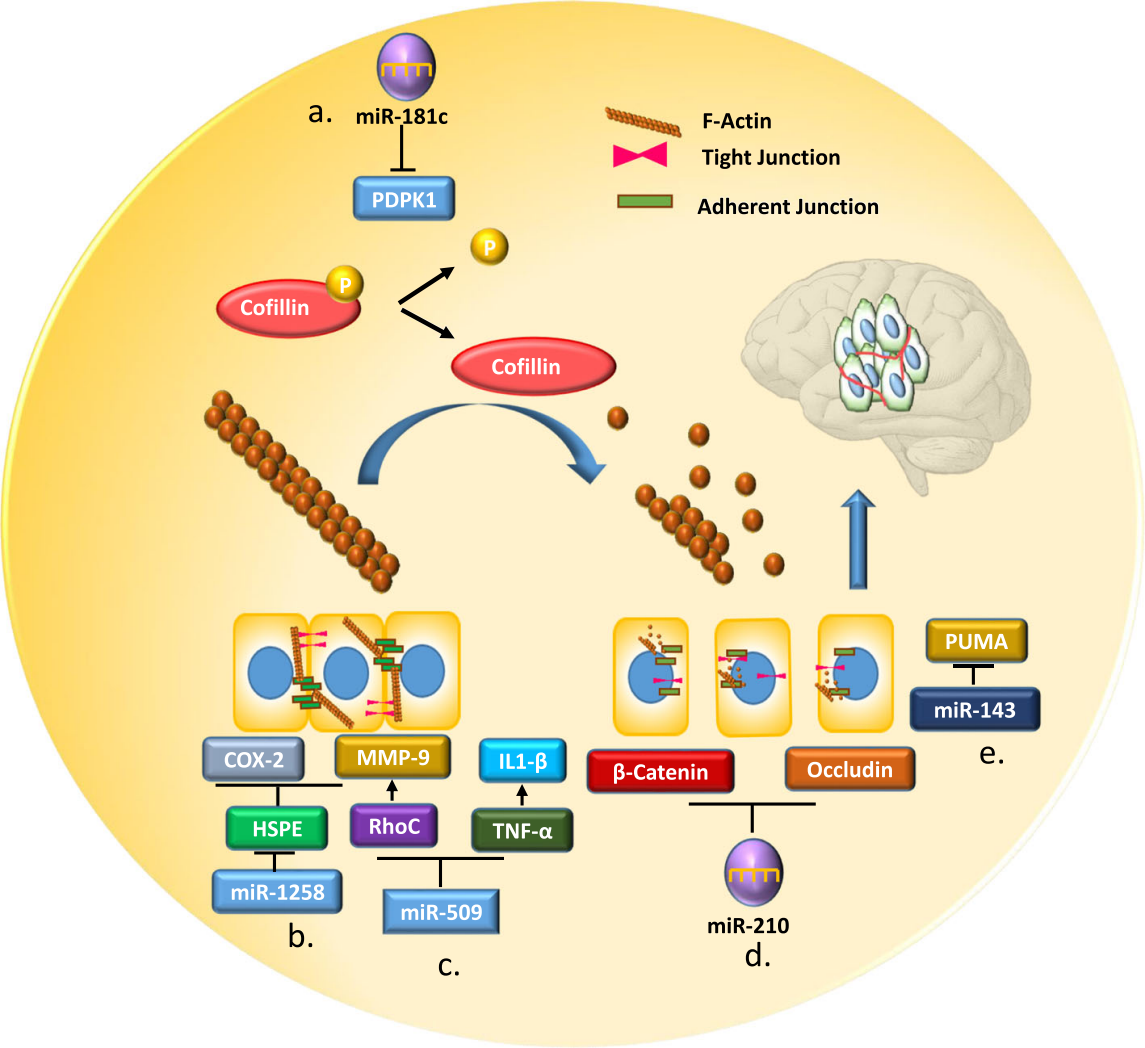
Schematic of miRNA regulatory blood-brain-barrier (BBB) tight junction (TJs) protein. **a** miR-181c promotes the destruction of the BBB through the delocalization of actin fibers via the downregulation of 3 phosphoinositide-dependent protein kinase-1 (PDPK1). PDPK1 degradation by miR-181c leads to the downregulation of phosphorylated cofilin and the resultant activated cofilin-induced modulation of actin dynamics [69]. **b** miR-1258 downregulates MMP-9 and COX-2 protein by directly targeting HPSE, hence protecting the BBB from destruction [40]. **c** miR-509 negatively regulates the expression of two essential genes for brain metastasis, RhoC and TNF-α, which enhance the permeability of the BBB [121]. **d** miR-210 directly targets β- Catenin and Occudin to disrupt the integrity of the BBB [37]. **e** MiR-143 decreases the expression of TJs by directly targeting p53 upregulated modulator of apoptosis (PUMA) and increases the permeability of human brain endothelial cells [35]

The priming of the pre-metastatic niche, or organotropism, starts before cancer cells reach the metastatic site from the primary tumors via paracrine routes. In this context, miRNAs containing exosomes or extracellular vehicles (EVs) have the ability to modify the brain microenvironment, which leads to enhanced BM despite the barrier function of the BBB [42, 120]. Recently, miRNAs emerged as regulators of tight junction adhesion proteins and their upstream and downstream signaling pathways, playing an important role in maintaining the integrity of the BBB. For instance, miR-181c promotes the destabilization of the BBB through the delocalization of actin fibers via the downregulation of 3-phosphoinositide-dependent protein kinase-1 (PDPK1). PDPK1 degradation by miR-181c leads to the downregulation of phosphorylated cofilin and a resultant activated cofilin-induced modulation of actin dynamics [69]. MiR-143 enhances the permeability of endothelial cells through targeting p53 upregulated modulator of apoptosis (PUMA), and consequently shows a reduction of tight junction proteins (TJPs) [35]. Additionally, miR-125a-5p has been shown to be an important player in the maintenance of the integrity of the BBB. This miRNA can directly regulate barrier function in an *in vitro* BBB model and can reduce monocyte migration through a BBB cell layer *in vitro* [38] (Fig. 1).

In BC, miR-1258 expression was directly associated with heparanase expression. Heparanase is a prometastatic enzyme present in BCBM cells that degrades heparan sulfate chains to affect the cytoskeleton and render cells more capable of crossing the BBB [39, 40]. Researchers demonstrated miR-1258 downregulates the phosphorylation of Akt and EGFR signaling along with the repression of MMP-9 and COX-2 protein expression by direct targeting of HPSE [39, 40]. Watabe K et al. observed high expression of miR-509 in primary tumors whereas level was significantly downregulated in BM lesions. Consequently, the reduction of miR-509 in BM lesions induces the expression of two essential genes for BM, RhoC and TNF-α, followed by upregulation of the MMP9 level, which altogether augments the permeability of BBB and penetration of tumor cells in the brain [121]. MiR-210 suppresses the junction proteins and disrupts the BBB in hypoxic-ischemic brain injury [37]. In addition, high expression of miR-210 is associated with poor survival in BC patients [36]. Exosomal profiling done by Dario et al. showed significant upregulation of miR-210 (2 to 6-fold increase) in three brain metastatic BC cell-derived exosomes [122]. Therefore, it is plausible that miR-210-containing exosomes released by the brain may help BC cells breach the BBB.

### Crosstalk of cancer cells with brain microenvironment

Once infiltrated into the brain tissue, cancer cells encounter a number of host cell types, including pericytes, reactive glia, neural progenitor cells, neurons, and oligodendrocytes [123]. Astrocytes and endothelial cells are the first to encounter incoming metastatic cells. Once normal astrocytes encounter cancer cells, they become reactive astrocytes (RAs) due to a perceived disruption to brain homeostasis. At the initial stages of BCBM, RAs act as a primary host defense system by proficiently limiting the survival of arriving metastatic cells [102], whereas, at later stages, RAs have been actively involved in promoting metastatic outgrowth via secretion of miRNA containing exosomes [42]. Exosomes can successfully form the pre-metastatic niche in the brain by modulating tumor-stroma communications [74]. MiRNAs with gene regulatory functions have emerged as key regulators of the tumor microenvironment [124]. For instance, miR-26a is present in astrocytes and released through exosomes or by endothelial cells. MiR-26 can regulate the growth of brain tumors and radiosensitize tumor cells by targeting PTEN and ATM, respectively [70, 71]. Thus, miR-26a may play a key role in regulating the brain tumor microenvironment.

Zhang et al. (2015) discovered a complicated reciprocal mechanism between brain metastatic BC cells and stromal cells (astrocytes and myeloid cells). In BCBM patients, PTEN loss was observed when compared to primary breast tumors. In addition, miR-19a in the miR-17-92 cluster was identified as a candidate responsible for mediating PTEN suppression from astrocytes to tumor cells via exosomes. Reactive astrocytes secrete interleukins and chemokines, such as CCL2 and CXCL12/SDF1, respectively, playing a mitogenic role. Moreover, human BCBM has higher levels of CCL2 than primary tumors. Interestingly, PTEN has been shown to be instrumental in the regulation of BCBM, as a reduced expression of PTEN leads to enhanced CCL2-mediated recruitment of IBA1^+^ microglial cells, and thereby establishment of the BM [42].

Astrocytes have also been shown to enhance metastatic growth through enhancing the CXCL12/CXCR4-MIR345-KISS1/KISS1R axis. A significant reduction in KISS1 expression in BCBM patient’s primary tumors has been noticed. Ilya V. Ulasov et al. identified that CXCL12 secreted by astrocytes can induce miRNAs that can directly target KISS1 mRNA in metastatic BC cells and negatively regulate KISS1 expression. In this regard, miR-345 was the only identified miR that directly targets KISS1, which is induced via the treatment of CXCL12 or CCL2 proteins [125–127]. They experimentally confirmed the binding of miR-345 in stably transfected KISS1 3’UTR in MDA231Br cells with astrocyte conditioned media treatment or in the presence of individual recombinant CCl2 or CXCL12 proteins [72]. Interestingly, the downregulation of KISS1 has a stimulating effect on ATG5 expression associated with autophagosome maturation. Finally, they revealed a paracrine loop between KISS1 and the CXCL12-miR-345 that can promote BC cell invasion and survival in the brain.

**Fig. 2.**
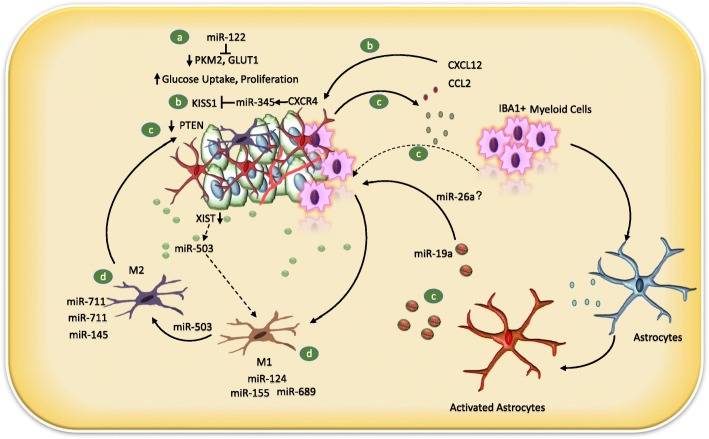
Cross talk of the brain tumor microenvironment with BC cells. **a** Autocrine and paracrine role of miR-122 in the development of the pre-metastatic niche via regulating glucose metabolism in cancer cells. MiR-122 downregulates the expression of pyruvate kinase isozymes, PKM2, and glucose transporter 1 (GLUT1), and decreases ATP levels in BC cells. MiR-122 reduces glucose consumption in stromal cells and allows more glucose to be accessible to cancer cells, hence facilitating the formation of the metastatic niche and cancer cell growth [74]. **b** CXCL12 or CCL2 secreted by astrocytes increases the level of miR-345 via CXCR4, which negatively regulates the expression of KISS1 and promotes invasion and survival in the brain [72]. **c** MiR-19a mediates the suppression of PTEN in cancer cells secreted by activated astrocytes. Reactive astrocytes secrete interleukins and chemokines, such as CCL2 and CXCL12/SDF1. Reduced expression of PTEN leads to enhanced CCL2-mediated recruitment of IBA1^+^ myeloid cells, and thereby establishment of the brain metastasis (BM) [42]. miR-26a is present in astrocytes and released by astrocytes through exosomes, or it can be secreted by HUVEC cells, but its role in brain niche formation is not clear [70, 71]. **d** Microglia release cytokines and interleukins that support cancer cells to invade and colonize the parenchyma. In cancer microglia, it can transform from the immunogenic phenotype (M1) to immunosuppressive phenotype and miRNA can modulate microglial polarization. MiR-124, miR-155, and miR-689 are associated with the M1 phenotype, whereas MiR-711 and miR-145 are strongly associated with M2 polarization [128, 129]. Loss of XIST, a long noncoding RNA in tumor cells, causes local immune suppression by converting the microglia to the M2 phenotype through the transport of exosomal miR-503 from the tumor cells [41]

Microglia are also a crucial component of the brain parenchyma; they constitute about 5-20% of the total CNS population and they are the only brain resident myeloid cells that play an important role in brain homeostasis and immunosurveillance [128–130]. Microglia release cytokines and interleukins that support cancer invasion and colonization of the parenchyma. Microglia can differentiate from the proinflammatory M1 phenotype to the immunosuppressive M2 phenotype based on environmental factors [131]. Interestingly, miRNA can modulate microglial polarization. For instance, miR-124, miR-155, and miR-689 are associated with the M1 phenotype, whereas miR-711 and miR-145 are strongly associated with M2 polarization [73]. MiR-124 is a brain enriched miRNA highly present in resting microglia, and its expression declines with microglial activation [132]. However, the role of miR-124 in BCBM is yet to be studied. Loss of XIST, a long noncoding RNA in tumor cells, causes local immune suppression by converting the microglia to the M2 phenotype through the transport of exosomal miR-503 from the tumor cells [41]. These studies strongly suggest that miRNAs have the ability to modulate microglia activation, and thereby modulate the brain microenvironment and subsequently metastasis partly via immune invasion (Fig. 2).

### miRNAs and metabolic reprogramming in the brain microenvironment

Adaptation in the pre-metastatic niche is of great importance and starts before the arrival of CTCs to distant sites of metastasis to sustain their survival and growth [133]. Modulation of the tumor microenvironment by metabolic factors is a different aspect of cancer cells and tumor microenvironment crosstalk. Metabolic reprogramming is associated with the deregulation of several pathways controlled by hypoxia-inducible factor 1 alpha, MYC, p53, and miRNAs. MiRNAs target metabolic enzymes, oncogenes, and tumor suppressors involved in metabolic reprogramming, becoming crucial elements in the crosstalk of molecular pathways that promote extravasation and metastasis. In BC, cancer-associated stromal cells rely on glycolysis to provide energy metabolites to cancer cells through monocarboxylate transporters during disease progression [134]. Endothelial cells also rely on glycolytic metabolism to support vessel sprouting for angiogenesis [134]. Emerging evidences in the metabolic reprogramming of the microenvironment identified a prerequisite metabolic condition required to sustain cancer cells in the brain [135]. For example, brain metastatic cells switch to metabolic reprogramming by upregulating the fructose-1, 6-bisphosphatases (FBP2) based gluconeogenesis pathway and amino acid oxidation to survive and grow in the low glucose environment of the brain parenchyma [135].

Interestingly, Emily Wang’s group has studied the autocrine and paracrine role of miR-122 in glucose metabolism in primary BC and pre-metastatic niche development and metastasis [74]. MiR-122 downregulates the expression of pyruvate kinase isozymes, PKM2, and glucose transporter 1 (GLUT1) and decreases ATP levels in BC cells. They demonstrated that cancer cells secreted miR-122, which downregulates glucose uptake in astrocytes as well as lung fibroblasts. Orthotopic xenograft mice with stably overexpressed DCISMCF/miR-122 form smaller tumors than empty vectors. Collectively, they showed that cancer cells could induce glucose reallocation in the pre-metastatic niche by repressing glucose consumption in stromal cells and allowing more glucose to be accessible to cancer cells, hence facilitating metastatic cancer growth. MiR-122 partially exhibits this effect and helps in metabolic reprogramming of the tumor microenvironment by downregulating its metabolic target genes PKM1/2 and GLUT1 in stromal cells *in vitro* and *in vivo.* MiR-122 has potential as a predictive marker and therapeutic target for BC metastasis [74].

Furthermore, Chang et al. recently demonstrated the role of miR-155 in glucose metabolism in the TNBC subtype. Utilizing a BC mouse model with miR-155^-/-^ or miR-155^+/-^ backgrounds, they unraveled the miR-155-PIK3R1-PDK/AKT-FOXO3a-cMYC axis that mediates energy metabolism in BC [75]. However, the metastatic potential of miR-155 has not been studied in the context of BM. High glucose uptake is a salient feature of cancer cells [136]. MiR-7 is highly expressed in the brain and promotes glycolysis, as evinced by an increased intracellular ATP/ADP ratio, glucose consumption, and lactic acid production. MiR-7 directly targets the expression of RelA, which regulates the expression of the cell surface glucose transporter, Glut3, hence promoting glycolysis [76, 137]. In human BC cells, miR-7 suppresses the homing and migration potential of human endothelial cells; however, there is the possibility that the opposite may be true and the brain tumor microenvironment may deliver exosomes containing miR-7 to increase the glucose uptake and survival of breast tumor cells in the brain parenchyma. Therefore, its role in the metabolic reprogramming of the brain microenvironment in BCBM is obscure and needs to be studied in detail.

### Metastatic colonization

The major problem with the EMT concept is that the appearance of the majority of human metastatic histology samples resembles the epithelial phenotype and usually looks like the primary tumor [138]. Evidence from previous studies suggests that for successful colonization and growth after extravasation to a secondary site to occur, cancer cells have to go through the mesenchymal to epithelial transition (MET) [138]. Interestingly, BCBM is dependent on cellular reprogramming through the EMT to the MET. Yang and colleagues (2012) have demonstrated that Twist1 reversibly regulates the EMT during metastasis. They have also shown that early metastatic colonies elicited strong positive Ki67 expression with low Twist1 expression under reversible EMT conditions, while irreversible EMT resulted in colonies with high Twist1 expression and low Ki67 [139], suggesting that metastatic cancer cells must revert to the epithelial phenotype by a MET in order to grow at a secondary site.

In another study, epithelial markers, such as E-cadherin, β-catenin, connexin 26, and connexin43, were found to be upregulated in BC patients. In contrast, mesenchymal markers FSP1 and vimentin were variably altered in BC, suggesting a partial MET [140]. Shreds of evidence show that miRNA participates in the process of EMT to MET [141]. A well-documented example is the miR-200 family. MiR-200s are associated with poor prognosis of BC [142]. Recently, members of the miR-200 family (miR-200a, miR-200b, miR-200c, miR-141, and miR-429, containing similar consensus seed sequence) have been recognized as new epithelial markers and negative regulators of EMT. The miR-200 family members inhibit the EMT and promote MET transformation in BC cells by directly targeting ZEB1 and ZEB2. The miR-200 family regulates the MET and metastatic colonization in BC, suggesting that flexible transitions between EMT and MET, or epithelial-mesenchymal plasticity, may be crucial at different stages of metastasis [43–45].

Moreover, human BC metastases often show a higher level of E-cadherin than their corresponding primary tumor [140]. Korpal et al. suggested that miR-200s promote metastatic colonization of BC not only by influencing cell-intrinsic epithelial traits through targeting of the Zeb–E-cadherin axis but also by altering the tumor cell-derived secretome through targeting of the Sec23 homolog A, Sec23a-mediated transport pathway. It ultimately targets two metastatic suppressors, insulin-like growth factor binding protein 4 (IGFBP4) and tubule interstitial nephritis antigen-like 1 [142].

**Fig. 3.**
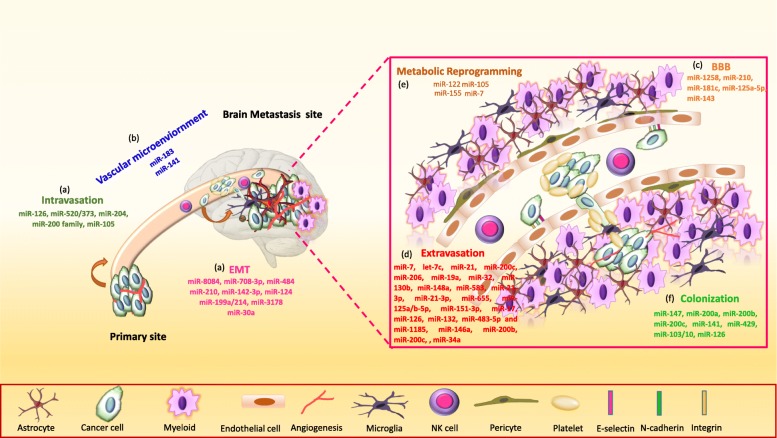
miRNAs function at multiple steps of breast cancer brain metastasis (BCBM). MiRNAs regulate key steps of BCBM, (**a**) breast cancer cell intravasation and dissemination via EMT from the primary site, (**b**) survival in the circulation/vascular microenvironment, (**c**) breaching of blood-brain barrier (BBB) integrity, (**d**) extravasation into brain parenchyma, (**e**) metabolic reprogramming into the brain microenvironment, and (**f**) colonization and growth of cancer cells into brain

In addition, CTCs increase the level of miR-200s in BC patient serum and cerebrospinal fluid (CSF) with BCBM [143, 144]. Although these studies suggest that extracellular miR-200s are associated with BC metastasis, they did not show that circulating miR-200 miRNAs are functional [145]. Bisrat G. Debeb et al. generated a preclinical mouse model via tail vein injection of epithelial-like inflammatory TNBC and HER2 positive cells and mesenchymal-like lung metastatic cells. The knockdown of miR-141 ceases the BM; however, ectopic expression of miR-141 enhances the brain colonization of inflammatory metastatic cells *in vivo*. Alternatively, ectopic expression of miR-141 in lung metastatic cells was not sufficient for the onset of BM, suggesting an epithelial phenotype is important at the final step of BM [31]. High expression of ZEB1 and ZEB2 at a tumor invasion front in brain metastatic tissues suggests a role of these EMT regulators in facilitating BM [146]. MiR-126 is reported as a tumor suppressor in various cancers [147–149]. It regulates the migration of endothelial cells towards the metastatic BC cells *in vitro* and *in vivo* [77]. Endogenous expression of miR-126 suppresses metastatic colonization by targeting IGFBP2, PITPNC1, and MERTK- novel pro-angiogenic genes and biomarkers of human metastasis [77]. Silencing of miR-126 in poorly metastatic CN34 BC cells results in increased endothelial recruitment and metastatic brain colonization [77]. Overall, the miRNAs are crucial at multiple steps of breast cancer brain metastasis (BCBM) (Fig. 3).

### miRNA and BCBM therapeutics

Despite advances in therapy for BCBM, the exact molecular mechanism, and biomarkers for the diagnosis and prognosis of patients are lacking [150]. Available treatment options include local therapies, such as whole-brain radiation therapy (WBRT), stereotactic radiosurgery (SRS), surgery, chemotherapy, and tyrosine kinase inhibitors (TKIs) [151]. TKIs are promising anticancer agents for HER2-positive BCBM, such as lapatinib, which is a dual TKI that targets both HER2/ErbB2 and EGFR. However, inhibition of kinases is not specific for a single tyrosine kinase, results in reduced specificity with high toxicity [152]. Due to specificity and toxicity issues, monoclonal antibodies emerged as strong therapeutic tools and proved as a potent therapy for cancer treatment. The current focus for TNBC patients is to manipulate the anti-tumor immune response by blocking the activity of immune checkpoint inhibitors. Atezolizumab, a PD-L1 blocking antibody in combination with nab-paclitaxel improved the progression-free survival in PD-L1 positive subgroup in an Impassion130 trial (NCT02425891) [153]. In phase II, ongoing trial (NCT03483012) TNBC patients with BM are treated with SRS alone or in combination with Atezolizumab. There is hope that SRS in combination with atezolizumab, may enhance the immune response to BM patients.

The enduring challenges in BCBM are to develop early detection markers and novel targeted therapies that can cross the BTB and improve the survival rate of BCBM patients. Therefore, miRNAs are emerging as noninvasive, diagnostic, and prognostic markers in BM. The miRNAs present in the blood plasma and CSF are attractive biomarkers for BM, which provide the disease severity, but also impart the prognostic value of the treatment response [154]. Although a considerable number of miRNAs are found inside the cell, many miRNAs are secretory, and their expression elevates or drops in the brain lesions or BM. In this context, miRNA levels can be used to monitor the disease burden, tumor response, and differentiation between brain lesions and metastatic brain tumors [155]. MiR-10 and miR-21 are highly expressed in the cases of GBM and BCBM; however, the miR-200 family can be used to discriminate between GBM and BM [144]. Additionally, miR-223, miR-711, miR-125, and miR-935 signatures were shown to discriminate among medulloblastoma, GBM, breast, and lung cancer BM [156].

Tumor suppressor miRNA with oncogenic targets may enhance the efficacy of treatment in combination with conventional chemotherapy, radiotherapy and immunotherapy in BCBM patients. For instance, miR-770-5p decreases the migration and invasive potential of HER+ breast cancer cells through inhibiting the translation of downstream signaling of PI3K and MAPK, i.e., AKT and ERK, pathways that mediate resistance to anti-HER2 therapies. Additionally, miR-770-5p can increase the responsiveness of trastuzumab and reverse drug resistance [157]. miR-770 also suppresses the doxorubicin-resistance and metastasis of TNBC cells [158, 159]. miR-326, a suppressor of Hedgehog pathway, is inversely correlated with multi drug resistance protein (MRP-1) expression in BC patients and sensitize the response in doxorubicin and etoposide (VP16) in resistant BC cells. miR-21 has been shown to sensitize BC cells to topotecan and taxol [160]. miRNA-143-3p increase the sensitivity of TNBC to paclitaxel by inferenig with CIAPIN1 expression, a cytokine-induced apoptosis inhibitor 1 protein [161]. miR-449 can induce doxorubicin respone in TNBC by downregulating cell cycle related genes [162]. Such combinations, which are already tested in BC preclinical models have the potential to be tested for BCBM. In addition, miRNA with known function in BM such as miR-181c, miR-1258, miR-509, miR-143, miR-122 and miR-19a could be utilized in combination with radiotherapy, anti-HER2 therapies (lapatinib or trastuzumab), chemotherapy or immunotherapies for BM.

Since miRNA can target multiple sets of genes, it is an excellent clinical choice for the heterogeneous population of BM. In this context, miR-7 has been shown to attenuate BC growth by downregulating both EGFR and PKB signaling pathways [156]. MiR-7 also inhibits BCBM by inhibiting the self-renewal capacity of BC stem-like cells by regulating the expression of KLF4 [163]. An additional example is let-7, which targets several oncogenic pathways, including Ras, HMGA2, cyclin d1/2/3, cyclin A, CDK4/6, c-Myc, DICER1, and Lin28, which are responsible for stem cell self-renewal and chemoresistance [164]. The current challenges with the delivery of miRNA into the brain are poor penetration of miRNAs into tumor tissues due to the presence of BBBs, instability of miRNA mimics or inhibitors in the blood circulation, and neurotoxicity and immunotoxicity due to an off-target effect. Therefore, miRNA can be conjugated to drug carrier systems or nanoparticles (NPs) for targeting cancer cells. These miRNA delivery systems have shown minimal toxicities and have the ability to cross the BBB and successfully release the miRNA to promote clinical advancement. Recently, numerous delivery systems have been developed to cross the BBB, such as Cationic lipid nanoparticles (LNP) [165], Cationic Dendrimers PAMAM [166], Poly (lactic acid-co-glycolic) acid (PLGA) nanoparticles [167], Magnetic Nanoparticles [168], and Viral Vector Systems [169, 170]. Although water-soluble polymers, cationic lipids, or liposome nanocarriers are less toxic than a viral vector, the delivery efficiency remains lower [171]. Since leukocytes (including monocytes/macrophages, neutrophils, dendritic cells, and lymphocytes) or MSCs target tumors and can migrate across physiological barriers like the BBB, these cell types are increasingly utilized as carriers to transfer NPs to tumors [172]. As leukocytes/MSCs follow the same pattern of migration as tumor cells to cross the BBB, these cellular mechanisms can be utilized effectively to deliver miRNA conjugated NPs to the BMs. These NPs can be attached to the monocytes/macrophages/MSCs for the delivery of miRNA through various nanotechnology strategies [173].

Additionally, BBB-permeable NPs can be used to deliver miRNAs into the metastatic sites. Recently, Galstyan et al. have used BBB-permeable nano-immunoconjugates for the successful inhibition of GBM growth using mouse models [174]. They have used poly (β-L-malic acid) NPs covalently attached to immune checkpoint antibodies for systemic delivery directly into the brain. NPs have been used to deliver anti-miR-132 that recover p120RasGAP in the tumor endothelial cells, and have shown reduction in tumor growth in an orthotopic xenograft mouse model of BC [175]. If BBB permeable NPs are not available, regular NPs containing miRNAs can be delivered through the BBB using chemical modifications, partial opening by ultrasound, microwave or electromagnetic field-based thermal translocation of tight junction proteins [176].

## Conclusion and future perspective

Both the anatomy and physiology of the brain are very complex; hence, the process of BCBM is enormously complex too. Mechanistic and functional discoveries could expedite the response of BM treatment. Each step of BM is rate-limiting, and miRNA are instrumental in the regulation of every step of metastasis since they are upstream of oncogenes and tumor suppressor genes. All the steps of BMs, starting from the dissociation from primary sites through EMT related genes, survival into the circulation by anoikis resistance genes, brain organotropism, brain niche modulatory genes, and also brain colonization related genes, are all regulated through miRNAs. In the past few years, the focus on BMs has significantly increased as several miRNAs were discovered for initiating steps of metastasis. However, limited research has been done to address questions like: how does miRNA play a role in metastasizing cancer cells to the brain? How can miRNAs breach the BBB? How do cancer cells communicate with an entirely new environment of the brain niche via miRNA? How do astrocytes overcome the defense mechanisms and facilitate the survival of BC cells by altering the miRNA profile? How do miRNAs modulate brain metabolism in favor of cancer cell survival? There are so many unanswered questions in the context of miRNA and BCBM. Therefore, intense research is needed to tackle these problems, to discover better treatment options, to improve BCBM treatment efficacy.

## Data Availability

Not applicable.

## Abbreviations

TNBC: Triple-negative breast cancer
HER2: Human epidermal growth factor receptor 2
BCBM: Breast cancer brain metastasis
ER: Estrogen receptor
PR: Progesterone receptor
TGF-β: Transforming growth factor-beta
TAFs: Tumor-associated fibroblasts
PAI-1: Plasminogen activator inhibitor-1
PTHrP: Parathyroid hormone-related protein
IL-8: Interleukin-8
IL-6: Interleukin-6
VEGF: Vascular endothelial growth factor receptor
NK cells: Natural killer cells
PDGF: Platelet-derived growth factor
PMPs: Platelet-derived microparticles
DAP-12: DNAX-activation protein 12
CTCs: Circulating tumor cells
PCDH7: Protocadherin-7
COX2: Cyclooxygenase-2
EGFR: Epidermal growth factor receptor
HBEGF: Heparin binding egf like growth factor
ST6GALNAC5: ST6N-Acetylgalactosaminide alpha-2,6-sialyltransferase
BBB: Blood-brain barrier
ZO-1: Zona occludens 1
PDPK1: 3- Phosphoinositide-dependent protein kinase-1
PUMA: p53 Upregulated modulator of apoptosis
TJPs: Tight junction proteins
RAs: Reactive astrocytes
PTEN: Phosphatase and tensin homolog
KISS1: KiSS-1 metastasis suppressor
XIST: X Inactive specific transcript
FBP2: Fructose-1,6-bisphosphatases
GLUT-1: Glucose transporter 1
PKM2: Pyruvate kinase isozymes
IGFBP4: Insulin-like growth factor binding protein 4
CDH1: Cadherin-1
